# Assembling the Marine Metagenome, One Cell at a Time

**DOI:** 10.1371/journal.pone.0005299

**Published:** 2009-04-23

**Authors:** Tanja Woyke, Gary Xie, Alex Copeland, José M. González, Cliff Han, Hajnalka Kiss, Jimmy H. Saw, Pavel Senin, Chi Yang, Sourav Chatterji, Jan-Fang Cheng, Jonathan A. Eisen, Michael E. Sieracki, Ramunas Stepanauskas

**Affiliations:** 1 DOE Joint Genome Institute, Walnut Creek, California, United States of America; 2 Los Alamos National Laboratory, Los Alamos, New Mexico, United States of America; 3 Department of Microbiology, University of La Laguna, La Laguna, Tenerife, Spain; 4 Department of Evolution and Ecology, University of California Davis, Davis, United States of America; 5 Bigelow Laboratory for Ocean Sciences, West Boothbay Harbor, Maine, United States of America; 6 Department of Microbiology, University of Hawaii at Manoa, Honolulu, Hawaii, United States of America; 7 Department of Information and Computer Sciences, University of Hawaii at Manoa, Honolulu, Hawaii, United States of America; 8 Institute of Bioinformatics, National Yang-Ming University, Taipei, Taiwan; University of Hyderabad, India

## Abstract

The difficulty associated with the cultivation of most microorganisms and the complexity of natural microbial assemblages, such as marine plankton or human microbiome, hinder genome reconstruction of representative taxa using cultivation or metagenomic approaches. Here we used an alternative, single cell sequencing approach to obtain high-quality genome assemblies of two uncultured, numerically significant marine microorganisms. We employed fluorescence-activated cell sorting and multiple displacement amplification to obtain hundreds of micrograms of genomic DNA from individual, uncultured cells of two marine flavobacteria from the Gulf of Maine that were phylogenetically distant from existing cultured strains. Shotgun sequencing and genome finishing yielded 1.9 Mbp in 17 contigs and 1.5 Mbp in 21 contigs for the two flavobacteria, with estimated genome recoveries of about 91% and 78%, respectively. Only 0.24% of the assembling sequences were contaminants and were removed from further analysis using rigorous quality control. In contrast to all cultured strains of marine flavobacteria, the two single cell genomes were excellent Global Ocean Sampling (GOS) metagenome fragment recruiters, demonstrating their numerical significance in the ocean. The geographic distribution of GOS recruits along the Northwest Atlantic coast coincided with ocean surface currents. Metabolic reconstruction indicated diverse potential energy sources, including biopolymer degradation, proteorhodopsin photometabolism, and hydrogen oxidation. Compared to cultured relatives, the two uncultured flavobacteria have small genome sizes, few non-coding nucleotides, and few paralogous genes, suggesting adaptations to narrow ecological niches. These features may have contributed to the abundance of the two taxa in specific regions of the ocean, and may have hindered their cultivation. We demonstrate the power of single cell DNA sequencing to generate reference genomes of uncultured taxa from a complex microbial community of marine bacterioplankton. A combination of single cell genomics and metagenomics enabled us to analyze the genome content, metabolic adaptations, and biogeography of these taxa.

## Introduction

The metabolism of bacteria and archaea drives most of the biogeochemical cycles on Earth [1], has a tremendous effect on human health [2], and constitutes a largely untapped source of novel natural products [3]. Recent advances in metagenomics revealed enormous diversity of previously unknown, uncultured microorganisms that predominate in the ocean, soil, deep subsurface, human body, and other environments [2], [4], [5], [6]. However, the recalcitrance to cultivation of the vast majority of environmental prokaryotes makes whole genome studies very challenging, if not impossible. Metagenomic sequencing of microbial communities enabled genome reconstruction of only the most abundant members [7], [8], [9]. While novel isolation approaches resulted in significant progress [10], [11], [12], they remain unsuited for high-throughput recovery of representative microbial taxa from their environment. The paucity of suitable reference genomes is a major obstacle in the interpretation of metagenomic data. For example, the first leg of the Global Ocean Sampling (GOS) expedition produced 6.3 Gbp of shotgun DNA sequence data from surface ocean microbial communities, but only a small fraction of the reads were closely related to known genomes, while no novel genomes were assembled [6]. These limitations of current methods in microbiology are illustrated by the difficulty in determining the predominant carriers of proteorhodopsins, which are abundant in marine metagenomic libraries and likely provide a significant source of energy to the ocean food web [6], [13], [14]. Thus, novel research tools are necessary to complement cultivation and metagenomics-based studies for the reconstruction of genomes, metabolic pathways, ecological niches, and evolutionary histories of microorganisms that are representative of complex environments.

To overcome current methodological limitations, we developed robust protocols for genomic sequencing from individual microbial cells. We used these novel tools to reconstruct genomes of two uncultured, proteorhodopsin-containing marine flavobacteria, MS024-2A and MS024-3C, which were isolated from the Gulf of Maine as previously described [15]. The 16S rRNA sequences of these two cells are distant from cultured strains, but closely related to several community PCR clones from diverse marine and Antarctic locations (Fig. S1). We demonstrate that, in contrast to their cultured relatives, these cells represent genetic material from numerically significant microbial taxa, which possess unique adaptations to the marine environment.

## Results and Discussion

### Single cell genome reconstruction

Shotgun sequencing and genome finishing resulted in 1.9 Mbp in 17 contigs and 1.5 Mbp in 21 contigs for the single amplified genomes (SAGs) MS024-2A and MS024-3C respectively, with contig length ranging 3–684 Kbp (Table 1). Based on the analysis of conserved single copy genes (CSCGs), these major contigs recovered about 91% and 78% of the two genomes (Figs. S2, S3, S4A, see Materials and Methods for more details). The uneven distribution of CSCGs on the genomes (Fig. S3) may introduce biases in this estimate. However, even considering such biases, complete genome sizes of MS024-2A and MS024-3C are likely to be within the relatively narrow ranges of 2.0–2.2 and 1.9–2.4 Mbp, respectively (Fig. S2B).

**Table 1:** General features of the single cell genome assemblies.

Assembly statistic	MS024-2A	MS024-3C
**Assembly size [Mbp]**	1.905	1.515
**Estimated genome size [Mbp]**	2.095	1.947
**Estimated genome recovery [%]**	91	78
**Number of contigs**	17	21
**Largest contig [kbp]**	684	549
**GC content [%]**	36	39
**Mean total read depth±sd**	56±63	83±110
**Mean 454 read depth**	47	68
**Mean Sanger read depth**	9	14.3
**Total genes**	1,815	1,413
**rRNA operons**	2	1
**tRNA genes**	33	24
**Protein-coding genes**	1,780	1,388
**Genes with no function prediction**	443	328

While 454 shotgun pyrosequencing provided lower-cost, high coverage depth data without cloning biases, the addition of paired-end Sanger sequencing assisted resolving homopolymer regions and improved genome assemblies (Fig. S4). Further shotgun sequencing would be ineffective due to the significant overrepresentation of certain genome regions in multiple displacement amplification (MDA) products (Fig. S5A).

On average, we detected one chimera per 13–27 Kbp of single cell whole genome MDA products (Table S1), which is comparable to prior reports [16], [17]. As single stranded DNA molecules represent intermediates in the chimera formation during MDA, S1 nuclease treatment has been suggested and shown to reduce chimerism [18]. We evaluated S1 nuclease mediated debranching effects by comparing 3 Kbp library clones from branched and unbranched MDA DNA for MS024-2A. No notable reduction in chimeric rearrangements was detected in the S1-treated DNA samples (Table S1). While the presence of MDA-produced chimera added a challenge to genome assembly and finishing efforts, sufficient sequencing coverage in most parts of the genomes allowed the identification and removal or chimeric reads from the assembly, as well as the identification of chimeric clones, which were avoided for primer walking.

Rigorous quality controls, using nucleotide pattern analysis and phylogenomics, were implemented to detect potential contaminants and amplification artifacts (Table S1, Figs. S5B, S6). Only 0.7% of all sequence reads and only 0.24% of the assembling reads were identified as contaminants or self-primed amplification products, and were removed from further analysis. In prior single cell genome sequencing attempts, Zhang et al. [18] recovered 66% of a genome of a cultured *Prochlorococcus* strain in 477 contigs, while Marcy et al. [19] recovered an unknown fraction of a genome of an uncultured representative of the TM7 phylum in 288 contigs, with up to 10% *Leptotrichia* contamination. Here we demonstrate that improved laboratory and bioinformatics protocols enable high-quality *de novo* draft reconstruction of genomes of uncultured taxa from complex microbial communities.

### Global Ocean Sampling (GOS) fragment recruitment

**Figure 1:**
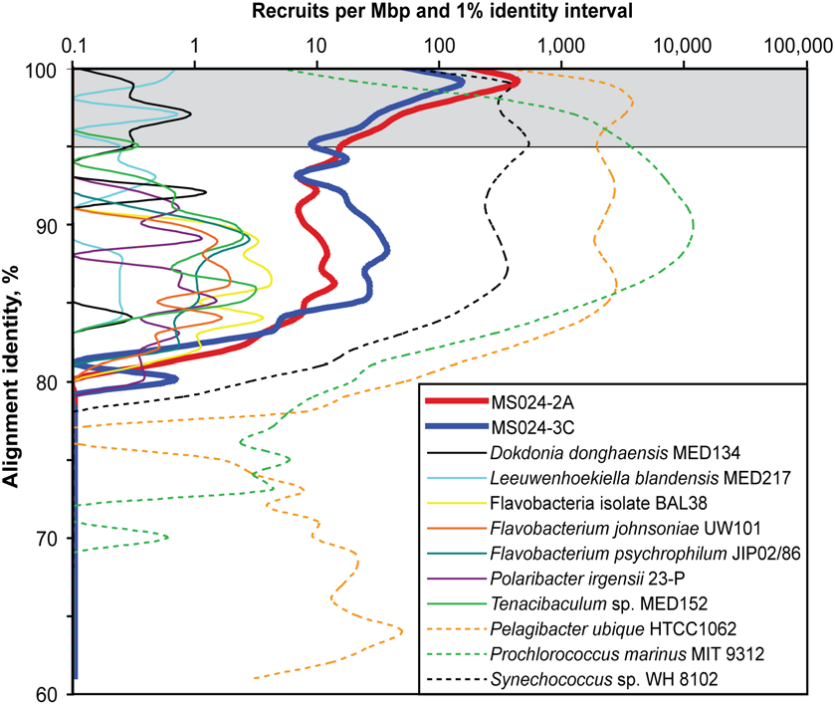
Global Ocean Sampling [6] metagenome fragment recruitment reveals SAGs MS024-2A and MS024-3C as strong recruiters. The available marine flavobacteria isolate genomes, the non-marine *Flavobacterium johnsoniae*, and the three best GOS fragment recruiters *Pelagibacter*, *Prochlorococcus* and *Synechococcus* served as reference genomes. Fragment recruitment was performed with MUMMER [55] and only ≥400 bp alignments were counted. *Psychroflexus torquis* ATCC 700755 was excluded from the analysis due to its poor genome assembly quality. The following marine flavobacteria genomes had fewer than 10 recruits and are not shown: *Croceibacter atlanticus* HTCC2559, *Robiginitalea biformata* HTCC2501, *Gramella forsetii* KT0803, *Kordia algicida* OT-1, isolate ALC-1, isolate HTCC2170, and isolate BBFL7. *Croceibacter atlanticus* HTCC2559 and *Robiginitalea biformata* HTCC2501 were originally collected at or near GOS sampling stations.

We searched for the presence of MS024-2A- and MS024-3C-like DNA in the (GOS) data using metagenome fragment recruitment [6]. The number of GOS fragments recruited by the two SAGs was higher, by at least one order of magnitude, than the recruitment by any of the eleven available genomes of cultured marine flavobacteria strains (Figs. 1, S7). The GOS read recruitment by marine cultures, including those collected at or near GOS stations, was as low as the recruitment by the soil isolate *Flavobacterium johnsoniae*. This suggests that currently sequenced flavobacteria cultures are poor representations of the predominant marine taxa, at least in the regions of the ocean represented by GOS data. In contrast, the number of recruits at high DNA identity level (>97%) was comparable for our two flavobacteria SAGs and the representatives of the ubiquitous marine genera *Prochlorococcus*, *Synechococcus*, and *Pelagibacter*, which were previously identified as the only significant GOS fragment recruiters [6]. This is quite remarkable, considering that the two SAGs are non-redundant genomes from a relatively small, pilot marine SAG library [15]. Our results demonstrate the power of single cell genomics to reconstruct representative microbial genomes from complex communities, independent of their cultivability.

**Figure 2:**
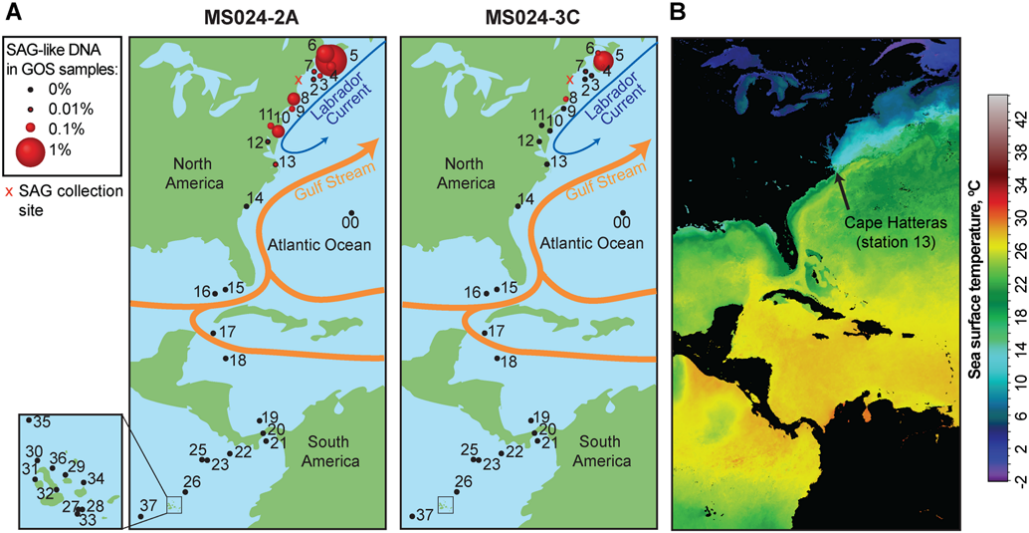
Biogeography of microorganisms closely related to MS024-2A and MS024-3C. A. Geographic distribution of the Global Ocean Sampling (GOS) metagenome fragments with >95% identity to MS024-2A and MS024-3C DNA. Numerals on the map indicate GOS station numbers. B. Sea surface temperature in December 2003, which demonstrates hydrological separation of GOS aquatic samples collected north and south of Cape Hatteras (near GOS station 13). Provided is a composite Aqua-MODIS image for December 2003 (http://oceancolor.gsfc.nasa.gov). The GOS stations were numbered in the order of their sampling, and stations 12, 13 and 14 were sampled on December 18, 19 and 20, 2003.

We further focused on the GOS recruits with >95% DNA identity to the two SAGs, as an operational demarcation of bacterial species [20]. A total of 1,505 and 467 of >95% DNA identity recruits were obtained for MS024-2A and MS024-3C. Of these, only nine recruits encoding only two genes were shared by the two SAGs, demonstrating significant evolutionary distance between the two genomes. Interestingly, >99% of the recruits and the two SAGs themselves came from a distinct biogeographic region along the coast of the northwest Atlantic Ocean (Fig. 2A). The fraction of SAG-like DNA did not correlate with ambient temperature, salinity, and chlorophyll *a* concentrations but was highest at the two northern-most GOS stations. In Bedford Basin, Nova Scotia (GOS station #5), 1.2% and 0.8% of all metagenomic reads were >95% identical to MS024-2A and MS024-3C DNA respectively, and in the Bay of Fundy (GOS station #6) 0.4% GOS reads matched to MS024-2A. No SAG recruits were found south of the GOS station #13 off Nags Head, North Carolina, including many tropical stations. This GOS recruit distribution correlates with the coastal transport of the remnants of the Labrador Current, as illustrated by the ocean surface temperature during the GOS sampling (Fig. 2B). It appears that close relatives of MS024-2A and MS024-3C are most abundant in the coastal northwest Atlantic waters and may be transported southward and mixed into local bacterioplankton assemblages by surface currents along the coastline. Single cells and the GOS Atlantic coast stations were sampled over 2 years apart (March 2006 and August-December 2003, respectively). Thus, MS024-2A and MS024-3C appear to represent two numerically significant marine flavobacteria taxa, which persist in particular geographic areas.

### Genome streamlining

The numerical significance of MS024-2A- and MS024-3C-like bacterioplankton in the intensely studied Atlantic coastal waters of U.S. and Canada raises two intriguing questions: 1) what makes these organisms competitive in their natural environment and 2) why are they not represented in cultures? Here we propose plausible explanations, as based on the SAGs' genome composition, including genome streamlining, energy-conserving metabolism, and diversified mixotrophy.

**Figure 3:**
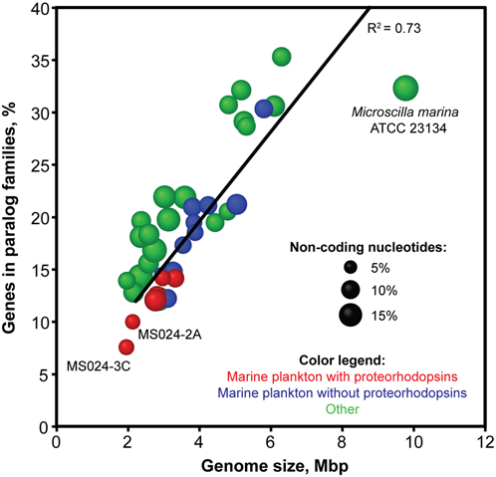
Genome streamlining in MS024-2A and MS024-3C. Genome streamlining was evidenced by small genome sizes, low fraction of genes in paralog families, and low fraction of non-coding bases. Included are all available genomes of the Bacteroidetes/Chlorobi group. The number of genes in paralog families was estimated using the BLASTCLUST tool from the NCBI BLAST software (>30% sequence similarity, across >50% of their length and E<10^−6^).

Genome streamlining was suggested as a nutrient and energy conserving adaptation in the ubiquitous and hard-to-culture marine alphaproteobacteria clade SAR11 [21]. Accordingly, MS024-2A and MS024-3C have among the smallest genomes, the lowest fraction of paralogous genes, and the lowest fraction of non-coding nucleotides amongst the sequenced taxa of the Bacteroidetes phylum (Fig. 3). The significantly reduced number of paralogs indicates that genome streamlining comes at a cost of reduced biochemical plasticity. Thus, MS024-2A and MS024-3C may represent taxa adapted to a narrow ecological niche, which may be one of the reasons behind their significant presence in a specific geographic area and difficulties in their laboratory cultivation.

Both MS024-2A and MS024-3C lack recognizable genes involved in the assimilation of sulfate, sulfite, nitrate or nitrite. The lack of nitrate and nitrite reductases is a common feature in all currently available Flavobacteria class genomes, while inorganic sulfur assimilation pathways appear to be missing in two sequenced flavobacteria isolates, *Flavobacterium psychrophilum* JIP02/86 and the isolate BAL38. Assuming the genome recovery of the two SAGs is 91% and 78% (Table 1; Figs. S2, S4), the probability for a single gene being missing from MS024-2A and MS024-3C due to the incomplete assemblies is 9% and 22%, respectively. Assuming that MDA bias and the resulting genome coverage by shotgun sequencing are random [16], the probability of a gene encoding the same metabolic function being missing from both SAGs due to incomplete genome recoveries is equal to 0.09×0.22 = 0.02, i.e. only 2%. With these qualifications, we hypothesize that the SAG-represented taxa rely solely on reduced N and S forms as an energy-saving strategy in organic C rather than inorganic N or S limited environment, as was recently described for “*Candidatus* Pelagibacter ubique” [21]. Experimental verification and field studies would be necessary to validate the inability for oxidized inorganic N and S utilization by some marine flavobacteria, as suggested by their genome features.

Although DMSP sulfur utilization was suggested by microautoradiography for a subset of marine flavobacteria in a community-level study [22], no significant homologs to known DMSP demethylases or lyases were detected on any of the available Flavobacteria class genomes, including these two SAGs. All available marine flavobacteria genomes also lack recognizable ureases, but most, including MS024-2A, encode allophanate hydrolases. Thus, allophanate, a breakdown product of urea, is a likely supplementary source of N to many marine flavobacteria. Both SAGs contain phosphate permeases and polyphosphate kinases, indicating their capacity for import and intracellular storage of inorganic phosphorus (Table S2).

### Proteorhodopsin photometabolism

The presence of proteorhodopsin genes (Flav2A_or1462, Flav3C_or0805) is yet another similarity between the two SAGs and the *Pelagibacter* genomes. Proteorhodopsins are light-driven proton pumps, which have recently been recognized for their abundance and likely biogeochemical significance in surface oceans [6], [13], [14]. However, the hosts of the majority of marine proteorhodopsins remain unidentified. Three recent studies, utilizing metagenomics, single cell genomics and cultivation, demonstrated the presence of proteorhodopsins in marine bacteroidetes [14], [15], [23]. Bacteroidetes-like proteorhodopsin genes are also abundant in diverse freshwater bacterioplankton communities [24]. Proteorhodopsin-containing microbial cultures currently include three alphaproteobacteria [21], [25], four bacteroidetes [23], and four SAR92 gammaproteobacteria [26]. Despite extensive tests, light stimulation likely attributable to proteorhodopsin activity has been detected in only one of these isolates, flavobacterium *Dokdonia* sp. MED134 [23]. Thus, the ecological roles and expression conditions of marine proteorhodopsins remain enigmatic.

Intriguingly, marine planktonic bacteroidetes with proteorhodopsins have smaller genomes and fewer paralogs compared to marine bacteroidetes without proteorhodopsins, while non-marine bacteroidetes have more paralogs and more non-coding DNA than their marine counterparts (Fig. 3; p<0.01, t-test). Although the causality of this relationship is unclear, the presence of proteorhodopsins in the streamlined genomes provides indirect evidence for their adaptive significance.

To examine proteorhodopsin relationships to other biochemical pathways, we investigated what genes are present in all six available proteorhodopsin-containing flavobacteria genomes but are absent in the remaining 13 flavobacteria genomes. Only three such genes were detected: proteorhodopsin, *blh* (encoding β-carotene dioxygenase, which produces proteorhodopsin chromophore retinal) and genes encoding DNA photolyase-like flavoproteins. The latter formed a distinct phylogenetic cluster among photolyase-like genes of flavobacteria (Fig. S8). It may be speculated that photolyase-like flavoproteins regulate rhodopsin proton pump expression or that both photometabolic systems are involved in synchronized photosensing or energy production. These hypotheses may be experimentally tested using pure cultures, metatranscriptome studies, or heterologous expression of SAG genes.

### Other metabolic features

Uniquely among marine flavobacteria, MS024-2A possesses [NiFe]-hydrogenase genes *hyaA* and *hyaB* (Flav2A_or1764, Flav2A_or1770), raising the possibility that this organism utilizes hydrogen as a supplementary source of energy. Potential sources of hydrogen in the ocean photic zone include photochemical reactions [27], algal metabolism [28], and heterotroph activity in anoxic microenvironments [29]. Hydrogenase-like genes are also harbored by the marine plankton *Roseobacter* clade isolates *Roseovarius* sp. HTCC2601, *Roseovarius* sp. TM1035 and *Sagittula stellata* E-37, and are abundant in GOS sequence data, which suggests a potentially widespread hydrogen metabolism in the ocean photic zone. The potential physiological and ecological significance of hydrogenases in marine bacterioplankton is intriguing and requires experimental verification.

Hydrogen oxidation and proteorhodopsin photometabolism may provide supplementary energy and a competitive advantage in a carbon-limited environment. However, the primary sources of carbon and energy for MS024-2A and MS024-3C likely are organic compounds. The two SAGs contain many genes involved in biopolymer hydrolysis (Table S3) and the import and degradation of hydrolysis products (Table S4). Both SAGs possess a substantial number of predicted proteins with domains that have been implicated in cell-surface and cell-cell interaction (Table S5). The characteristic repetitive domain structures in adhesion proteins are known to bind calcium ions, such as *Cadherin*, *FG-GAP* and *Thrombospondin type 3* repeats; or to bind cell receptors and metal ions, such as *Fasciclin* and *Von Willebrand factor type A*. These cell surface repetitive structures could play an important role in adhering to algal surface mucilage, in attaching to the nutrient-rich marine snow particles, and in biofilm formation. These features are consistent with the genome composition of other marine flavobacteria [30], with the community-level evidence of marine flavobacteria proficiency in biopolymer hydrolysis [31], and with the relative abundance of flavobacteria in algal blooms and in physical associations with algal cells - the likely sources of these biopolymers [31].

In contrast to all currently available Flavobacteria class genomes, MS024-2A contains an anti-sigma factor *rsbW*, its antagonist *rsbV*, an associated gene *rsbU*, and a PAS domain S-box (Fig. S9). The MS024-3C genome also contains *rsbW* and a fragment of *rsbU* at the end of a contig, while other genes of the operon are missing, possibly due to the incomplete MS024-3C assembly. It is likely that the *rsbW* cluster is involved in the global cellular response to changing environmental conditions, as in the model organism *Bacillus subtilis* with homologous genes [32].

### Conclusions

We demonstrate the power of single cell DNA sequencing to generate representative reference genomes of uncultured taxa from a complex community of marine bacterioplankton. A combination of single cell genomics and metagenomics enabled us to analyze the genome content, metabolic adaptations, and biogeography of numerically significant, uncultured microorganisms.

## Materials and Methods

### Environmental sample collection, cell sorting, and first round of whole genome amplification

Coastal water sample was collected from Boothbay Harbor, Maine, from 1 m depth at the Bigelow Laboratory dock (43°50′39.87″N 69°38′27.49″W) on March 28, 2006. Bacterioplankton were stained with a generic live-cell DNA stain SYTO-9 (Invitrogen), and individual, high nucleic acid cells were selected at random and sorted into 96-well plates using a MoFlo™ (Dako-Cytomation) flow cytometer, as previously described [15]. Protocols for single cell lysis, whole genome MDA, and PCR-based screening have been described previously [15]. Of the eleven marine single amplified genomes (SAGs) obtained from this sample, two were identified as proteorhodopsin-containing flavobacteria [15]. The original MDA products of these two SAGs, named MS024-2A and MS024-3C, were re-amplified using REPLI-g MIDI kit (Qiagen) following manufacturer's instructions. To minimize biases of the second MDA reaction, 14 replicate 100 µL reactions were performed and then pooled together, resulting in 700–800 µg of genomic dsDNA from each SAG. MDA products were debranched using S1 nuclease (Fermentas) digestion with 10 U/µl at 37°C for 1 h. The enzyme was heat-inactivated in the presence of EDTA and the DNA was phenol-chloroform-isoamyl alcohol extracted and ethanol-precipitated.

### 16S rRNA clone libraries

Bacterial 16S rDNA PCR libraries were created from debranched MDA products using primers 27f and 1391r [33]. Ribosomal RNA gene PCR amplification using universal archaeal 16S primers as well as eukaryotic 18S primers was attempted but did not yield any PCR products. PCR amplicons of five replicate reactions were combined and ligated into the pCR4-TOPO vector using the TOPO TA Cloning Kit (Invitrogen). Ligations were then electroporated into One Shot TOP10 Electrocomp™ *E. coli* cells and plated on selective media agar plates. The bi-directional 16S rDNA sequence reads were end-paired, trimmed for PCR primer sequence and quality and analyzed using BLASTn [34]. Three out of 332 16S rDNA clone sequences were not identical to the MS024-2A 16S gene. Based on their phylogeny (one *Pseudomonas* and two Crenarchaea), the three clones were most likely introduced as contaminants during the cloning/sequencing process. For flavobacteria bacterium MS024-3C, all 267 16S rDNA clone sequences were target-specific, suggestive of MDA product purity. Previously, 16S rRNA fingerprinting analysis by terminal restriction fragment length polymorphism (T-RFLP) inferred that the flavobacterial SAGs MS024-2A and MS024-3C are lacking evident contamination [15].

### Genome sequencing

A combination of Sanger shotgun sequencing and 454 pyrosequencing was performed on the single cell MDA products. For Sanger sequencing, 3 Kbp and 8 Kbp shotgun libraries were constructed using debranched MDA products. To evaluate the debranching effects, an additional 3 Kbp library was constructed using untreated MDA products of MS024-2A. For shotgun library construction, MDA products were randomly sheared to 2–4 Kbp and 6–10 Kbp fragments using HydroShear (GeneMachines). The sheared DNA was separated on an agarose gel, gel-purified using the QIAquick Gel Extraction Kit (Qiagen) and blunt-ended using T4 DNA polymerase (Roche) and Klenow Fragment (New England Biolabs) in the presence of dNTPs and NEB2 buffer. The 2–4 Kbp and 6–10 Kbp DNA fragments were ligated in pUC19 vector (Fermentas) and pMCL200 vector, respectively, O/N at 16°C using T4 DNA ligase (Roche Applied Science) and 4.5% polyethylene glycol (Sigma). The ligation products were phenol-chloroform extracted and ethanol precipitated. According to the manufacturer's instructions, ligations were electroporated into ElectroMAX DH10B™ Cells (Invitrogen) and clones prepared and sequenced on an ABI PRISM 3730 capillary DNA sequencer (Applied Biosystems) according to the JGI standard protocols (www.jgi.doe.gov). End-sequencing yielded 7,680 reads (totaling 4.58 Mbp) of 3 Kbp clone sequence and 19,968 reads (totaling 12.87 Mbp) of 8 Kbp clone sequence for MS024-2A. We generated 7,680 3 Kbp library reads (totaling 5.05 Mbp) and 29,952 8 Kbp library clones (totaling 17.67 Mbp) for MS024-3C. Pyrosequencing was performed on debranched MDA products using the Genome Sequencer FLX System (454 Life Sciences, http://www.454.com/) [35] according to the manufacturer protocol. The sequencing runs generated ∼95 Mbp (MS024-2A) and 108 Mbp (MS024-3C).

### Tetramer analysis

To detect possible DNA contamination, we designed a novel test using oligonucleotide frequencies, similar to CompostBin [36]. The frequencies of tetramers were extracted from each Sanger sequence read and used to represent the data as a N×256 feature matrix, where N is the number of sequence reads and each column of the matrix corresponds to the frequency of one of the 256 possible tetramers. Principal Component Analysis [37] was then used to extract the most important components of this high dimensional feature matrix and the projections into the first two Principal Components were analyzed as based on their modality and visualized in a scatter plot. The Matlab and C code for the oligonucleotide tests are available freely at the website http://bobcat.genomecenter.ucdavis.edu/souravc/singlecell/. Lastly, the Sanger reads were analyzed by blastx [34] against the Genbank nr database. Reads were taxonomically assigned using MEGAN [38].

### Genome assembly and finishing

The pyrosequence reads were assembled using the 454 Newbler assembler version 1.1.02.15 and the consensus sequence shredded into 2 Kbp pieces with 100 bp overlaps. The 454 shred data was assembled with the Sanger sequences using parallel Phrap (High Performance Software, LLC). The Phred/Phrap/Consed software package (www.phrap.com) was used for sequence assembly and quality assessment [39], [40]. Chimeric reads were detected and excluded from the assemblies using local Perl scripts. Possible mis-assemblies were corrected with Dupfinisher [41] and manual editing. To close the gaps and to raise the quality of the sequences, primer walking on the medium and small insert size clones, and PCR/adapter PCR [42] on the MDA products were performed. A total of 4,494 primer walk reads and 220 PCR/adapter PCR reads were generated during finishing for MS024-2A. For MS024-3C, we generated a total of 2,076 primer walk reads and 197 PCR/adapter PCR reads. The smallest two MS024-2A contigs (∼0.24% of the assembly) were identified as contamination as based on GC-content, tetramer binning, and BLAST analysis, and were thus excluded from this draft genome. Final assembly sizes were 1,905,484 bp (17 contigs) for MS024-2A and 1,515,248 bp (21 contigs) for MS024-3C.

**Estimates of complete genome sizes** were obtained for MS024-2A and MS024-3C using conserved single copy gene (CSCG) analysis. To identify relevant CSCGs, 16 Flavobacteria class genomes, currently available at the Joint Genome Institute Integrated Microbial Genomes site (IMG; http://img.jgi.doe.gov/cgi-bin/pub/main.cgi) [43], were included in the analysis: *Capnocytophaga ochracea* DSM 7271, *Dokdonia* sp. MED134, *Croceibacter atlanticus* HTCC2559, Flavobacteria bacterium BAL38, Flavobacteria bacterium BBFL7, Flavobacteriales bacterium ALC-1, Flavobacteriales bacterium HTCC2170, *Flavobacterium johnsoniae* UW101, *Flavobacterium psychrophilum* JIP02/86, *Gramella forsetii* KT0803, *Kordia algicida* OT-1, *Leeuwenhoekiella blandensis* MED217, *Polaribacter irgensii* 23-P, *Robiginitalea biformata* HTCC2501, *Polaribacter* sp. MED152, and *Ulvibacter* sp. SCB49. The genome of *Psychroflexus torquis* ATCC 700755 was excluded due to its poor assembly quality. The pre-computed COG function distribution from the 16 genomes was retrieved from IMG using Function Profile feature. First, one of the 16 genomes was randomly selected as a “seed” to quantify single copy genes. Then additional, randomly selected genomes were sequentially added, until all 16 genomes were included, and the number of CSCGs, i.e. single copy genes shared among the analyzed group of genomes, was quantified for each genome combination. The entire process was reiterated 1000 times, using new, randomly selected “seed” genomes. In this way we identified 268 CSCGs that were shared by all 16 available Flavobacteria class genomes (Fig. S2A). The number of CSCGs was plotted against the number of genomes analyzed, and a power function fit was applied to the data. We extrapolated this regression curve to predict that the number of CSCGs remaining after adding one more, 17th genome is 265, i.e. 6 genes (2.2%) fewer than with only 16 genomes involved. Of the 268 identified CSCGs, 239 (89%) and 204 (76%) were present on the assemblies of MS024-2A and MS024-3C. We used this information to estimate the expected complete genome sizes of MS024-2A and MS024-3C as follows:
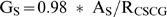
where G_S_ is the expected complete genome size; A_S_ is the size of current genome assemblies (1.9 Mbp and 1.5 Mbp for MS024-2A and MS024-3C); R_CSCG_ is the recovery of CSCGs (0.89 and 0.76 for MS024-2A and MS024-3C); and 0.98 is the correction coefficient to compensate for the expected lower number of CSCGs shared by 17 relative to 16 genomes. The application of this model resulted in the expected complete genome sizes of MS024-2A and MS024-3C to be 2.1 Mbp and 1.9 Mbp.

Many CSCGs are arranged in clusters (Fig. S3), which may lead to biases in the CSCG-based genome size estimates. For example, preferential recovery of CSCG-rich regions would lead to an underestimate of the genome size, and vice versa. To evaluate these potential biases, we used the three closed Flavobacteria class genomes as references: *Gramella forsetii* KT0803, *Flavobacterium johnsoniae* UW101 and *Flavobacterium psychrophilum* JIP02/86. Each of these genomes was sequentially divided into various numbers of equal-sized segments, from 1 to 360 segments per genome. The segmentation was repeated 18 times for each genome, by rotating the segmentation at 20° increments. Genome sizes of SAGs were then estimated based on the recovery of genes representing each of these reference genome segments, as follows:
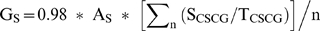
where G_S_ is the expected complete genome size; T_CSCG_ is the total number of CSCGs in a given reference genome segment; S_CSCG_ is the number of those genes recovered in a SAG, n is the total number of segments, and 0.98 is the correction coefficient to compensate for the expected lower number of CSCGs shared by 17 relative to 16 genomes. Genome size estimates varied somewhat, depending on the reference genome, the number of segments, and, to a lesser extent, rotation of the segmentation (Fig. S2B). The segmentation-based genome size estimates for MS024-2A and MS024-3C were 2.0–2.2 Mbp and 1.9–2.4 Mbp.

### Genome annotation and comparative analysis

Automated gene prediction was performed by JGI-ORNL using the output of Critica [44] complemented with the output of Generation and Glimmer [45]. The tRNAScanSE tool [46] was used to find tRNA genes, whereas ribosomal RNAs were found by using BLASTn vs. the 16S and 23S ribosomal RNA databases. Other “standard” structural RNAs (e.g., 5S rRNA, rnpB, tmRNA, SRP RNA) were found by using covariance models with the internal search tool [47]. The assignment of product descriptions was made by using search results of the following curated databases in this order: TIGRFam; PRIAM (E<10^−30^ cutoff); Pfam; Smart; COGs; Swissprot/TrEMBL (SPTR); and KEGG. If there was no significant similarity to any protein in another organism, it was described as “hypothetical protein”. “Conserved hypothetical protein” was used if at least one match was found to a hypothetical protein in another organism. EC numbering was based on searches in PRIAM at E<10^−10^ cutoff; COG and KEGG functional classifications were based on homology searches in the respective databases.

Comparative analyses were performed using a set of tools available in The Joint Genome Institute Integrated Microbial Genomes (IMG; http://img.jgi.doe.gov/cgi-bin/pub/main.cgi) [43]. Unique and orthologous MS024-2A and MS024-3C genes were identified by using BLASTp (cutoff scores of E<10^−2^ and 20% identity and reciprocal hits with cutoffs of E<10^−5^ and 30%, respectively). Signal peptides and transmembrane helices were predicted with SignalP 3.0 [48] and TMHMM 2.0 [49] set at default values. Protein localizations were predicted with PSORTb [50] and twin-arginine translocation systems were identified using TatP program [51]. Insertion sequence (IS) elements were identified using the ISFinder database [52]. Metabolic pathways were constructed with MetaCyc as a reference [53].

The sequence data has been deposited in GenBank (http://www.ncbi.nlm.nih.gov/Genbank) under project accessions ABVV00000000 (MS024-2A) and ABVW00000000 (MS024-3C).

### Metagenome fragment recruitment

Sequence reads from the Global Ocean Sampling (GOS) expedition were downloaded from the Camera website (http://camera.calit2.net/about-camera/full_datasets.php). Microbial isolate genome sequences were downloaded from NCBI website (http://www.ncbi.nlm.nih.gov/genomes/lproks.cgi). Contigs from the two flavobacteria MS024-2A and MS024-3C and selected isolate genomes were used as reference sequences and aligned against sequence reads from the GOS data using MUMmer [45], [54] with the following parameters: nucmer -minmatch 10 -breaklen 400 -maxgap 400 -mincluster 400. The ≥400 bp threshold for alignments was introduced to ensure that fragment recruitment is based on nucleotide homology of at least half of an average-length microbial gene. The fragment recruitment criteria used in this study were more stringent than those applied by Rusch et al. [6], resulting in the overall lower recruitment numbers in our study, compared to those reported by Rusch et al. [6] for the same reference genomes. Coordinate files produced from MUMmer alignments were parsed using an in-house developed Java program and alignment plots of the GOS reads against the reference sequences were created using an R script.

## Supporting Information

Figure S1 Maximum likelihood phylogenetic tree of 16S rRNA genes. Included are Flavobacteria isolates undergoing whole genome sequencing, single amplified genomes (SAGs) from the same environmental sample as MS024-2A and MS024-3C, as well as those sequences in Genbank that are ≥97% identical to MS024-2A and MS024-3C. Black circles indicate ≥70% neighbor-joining bootstrap support.
(0.31 MB TIF)

Figure S2 Genome size estimates. A: Number of conserved single copy genes (CSCGs) in Flavobacteria family genomes. The pre-computed COG function distribution from 16 genomes was retrieved from the IMG database, and the number of SCSGs was calculated through an iterative re-sampling of these genomes (see Materials and Methods). Provided are means and standard deviations. A power function fit was applied to the relationship between the count of genomes and the count of CSCGs. This function fit was extrapolated to predict the number of SCGS if a 17th genome was added. B: The effect of CSCG clustering in reference genomes on the estimates of MS024-2A and MS024-3C genome sizes. The genomes of *Gramella forsetii* KT0803, *Flavobacterium johnsoniae* UW101 and *Flavobacterium psychrophilum* JIP02/86 were sequentially divided into various numbers of equal-sized segments, from 1 to 360 per genome. The segmentation was repeated 18 times for each genome, by rotating the segmentation at 20° increments. Genome sizes of SAGs were then estimated based on the recovery of genes representing each of these reference genome segments, as follows: [Σ_n_(SCSCG/TCSCG)] * 0.98/n where TCSCG is the total number of CSCGs in a given reference genome segment; SCSCG is the number of those genes recovered in a SAG, n is the total number of segments, and 0.98 is the correction coefficient to compensate for the expected lower number of CSCGs shared by 17 relative to 16 genomes (Fig. S2A). Provided are means and standard deviations for estimates prepared by rotating the reference genome at 20° increments.
(1.89 MB TIF)

Figure S3 Locations of 268 single copy genes, conserved among Flavobacteria (CSCGs), on the genomes of *Gramella forsetii* KT0803 (A), *Flavobacterium johnsoniae* UW101 (B), and *Flavobacterium psychrophilum* JIP02/86 (C). The outermost two circles indicate start sites of genes and assigned functional categories: forward-strand gene products (circle 1) and reverse-strand gene products (circle 2). Circle 3 indicates RNA genes (tRNAs green, sRNAs red, other RNAs black); circle 4 indicates CSCGs; circle 5 indicates G+C content; circle 6 indicates GC skew (G−C/G+C, khaki are values >1, purple are values <1). Colors of the two outermost circles represent the following functional categories: amino acid biosynthesis, cyan; biosynthesis of cofactors, brown; cell envelope, light gray; cellular processes, light blue; central intermediary metabolism, dark salmon; energy metabolism, green; fatty acid and phospholipid metabolism, orange; other categories, salmon; protein fate, dark gray; purines, pyrimidines, nucleosides, and nucleotides, light green; regulatory functions, light salmon; replication, blue; transcription and translation, magenta; transport and binding proteins, yellow; unassigned, black; unknown function, red.
(1.76 MB TIF)

Figure S4 MS024-2A and MS024-3C genome coverage as a function of the sequencing effort (A); and the impact of 454 and Sanger sequence on the number of contigs (B), assembly size (C) and the number of scaffolds (D) for MS024-2A. For panel A, genome size estimates were based on conserved single copy gene (CSCG) analysis (see Materials and Methods). The curves display near-saturation, indicating that additional sequencing would mostly result in repeated sampling of the over-amplified genomic regions and not target the yet missing regions of the genomes. PCR amplification allowed the recovery of some of the missing sequence, suggestive of the under-representation but not lack of these regions. For panels B–D, up to ∼5 Mbp of 3 Kbp library Sanger sequence and ∼13 Mbp of 8 Kbp library Sanger sequence were randomly selected and assembled with 0–60 Mbp of randomly selected pyrosequence to evaluate the assembly outcome. Triangles represent 3 Kbp library sequence and circles represent 8 Kbp library sequence. Our data suggests that increased paired-end Sanger sequence reduced the number of contigs, bringing the assembly together. There was no apparent difference in the impact of 3 Kbp and 8 Kbp clones on the assembly. Adding 454 sequences to the Sanger reads was highly beneficial. Increasing amounts of 454 sequences (most notable at 60 Mbps) raised the number of contigs, which is likely attributed to the biased DNA representation: high amount of pyrosequence begins uncovering some of the under-represented genome regions, creating new contigs. The number of scaffolds kept rising with any added sequence, which is likely attributed to insufficient coverage. The data in panels B–D is based on uncurated pga (v2.6.2) assemblies. The assembly size in panel C thus differs from the extensively curated MS024-2A draft genome assembly size in panel A and Table 1.
(0.93 MB TIF)

Figure S5 Multiple displacement amplification bias (A) and GC content (B) in MS024-2A and MS024-3C shotgun sequence products. Significant MDA bias is evident from the sequence depth distribution plots for MS024-2A and MS024-3C (A). The contigs for the SAGs were aligned by length and contig breaks, indicated by the tic marks along each top panel. The GC content of the MS024-2A and MS024-3C Sanger sequence and pyrosequence reads demonstrate tight, unimodal distribution at 36% and 39%, suggesting that the reads originate from single phylotypes (B). Contamination with genomes of the same GC contents would, however, be undetectable.
(1.22 MB TIF)

Figure S6 Principal component analysis of nucleotide tetramer frequency in Sanger reads of MS024-2A (A) and MS024-3C (B). The taxonomic origins of the reads were inferred by blastx against GenBank nr database and summarized by MEGAN. Outliers I (0.7% of all reads) were identified as proteobacterial contamination, which may have been introduced during the sequencing process. Outliers II (0.02% of all reads) were identified as polyAT sequence, possibly derived through a buildup of random hexamers. Using simulated datasets from different species of the same GC contents, we were able to separate the genome sequences as based on tetramer signatures (data not shown). The integration of tetramer frequency, blast, and GC content analyses enabled accurate detection of the low levels of contaminating DNA in the shotgun libraries.
(1.82 MB TIF)

Figure S7 Global Ocean Sampling metagenome fragment recruitment by MS024-2A and MS024-3C and the three best GOS fragment recruiters: *Synechococcus* sp. WH8102, *Prochlorococcus marinus* strain MIT 9312 and 9312 and “Candidatus Pelagibacter ubique” HTCC1062. Fragment recruitment was performed with MUMMER and only ≥400 bp alignments were counted. For the two SAGs, the contigs are arranged by length along the x-axis, as indicated by the tick marks.
(1.58 MB TIF)

Figure S8 Neighbor-joining tree of DNA photolyase-like genes from all available Flavobacteria genomes. Indicated are IMG gene object identifiers and strain or SAG names. Colors represent marine organisms with rhodopsins (red), marine organisms without rhodopsins (blue), and non-marine organisms (green).
(0.40 MB TIF)

Figure S9 The structure of the *rsb* operon in MS024-2A (A) and a model for the regulation of σ^24^ in MS024-2A (B). In panel B, σ^24^ is held inactive in unstressed MS024-2A as a complex with an anti-sigma factor RsbW. The σ^24^ is freed from RsbW when a release factor, RsbV, binds to RsbW. In other words, RsbW forms mutually exclusive complexes with either the RsbV protein or σ^24^. In an unstressed cell, RsbV is inactive due to an RsbW-catalyzed phosphorylation (RsbV-P). Physical stress activates an RsbV-P phosphatase RsbU, which reactivates RsbV. Upon exposure to stress, the putative transmembrane RsbU phosphatase is activated either by a signal at its N-terminal domain or PAS sensory domains of upstream adenylate cyclase.
(0.40 MB TIF)

Table S1 Chimeric rearrangements in SAG DNA. To identify chimeric reads and clones, reads were Q20 quality trimmed and Blast-aligned against the SAG draft assemblies with an alignment minimum of 25 bp. On average, we detected one chimera per 13–27 Kbp of single cell whole genome multiple displacement amplification products. No notable reduction in chimeric rearrangements was detected in the S1-treated DNA samples.
(1.27 MB PDF)

Table S2 Key enzymes and metabolic pathways in the uptake and metabolism of N, P, S and Fe. Two pathways for ammonium assimilation were detected in MS024-3C, Gln synthetase (GS)/Glu synthase (GOGAT), and Glu dehydrogenase (GDH) pathways. MS024-2A only contains the GDH pathway. Genes involved in nitrate or nitrite utilization were not found. Polyphosphate kinase catalyzes the formation of polyphosphate granules from ATP. H^+^-translocating pyrophosphatase couples the energy of PPi hydrolysis to H^+^ movement across the membrane. Polyphosphate is also a source of PPi. Sulfate assimilation genes involved in the reduction of sulfate to H2S (*cysDHNIJE*) could not be detected. The sole source of sulfur appears to be organic material or H_2_S. Most of the peptides involved in iron metabolism are transporters. TonB-dependent outer membrane channels are mainly known for the transport of iron in Gram-negative bacteria.
(0.07 MB PDF)

Table S3 The total and genome size (Mbp)-normalized number of hydrolytic enzymes, TonB dependent/ligand-gated channels and SusD homologs. Included in the analysis are MS024-2A, MS024-3C and other Bacteroidetes genomes with high hydrolytic potential (*Flavobacterium johnsoniae* UW101, *Gramella forsetii* KT0803, *Cytophaga hutchinsonii* ATCC 33406, *Bacteroides thetaiotaomicron* VPI-5482, *Flavobacterium psychrophilum* JIP02/86) and proteorhodopsin-containing Bacteroidetes (*Polaribacter* sp. MED152, *Polaribacter irgensii* 23-P, *Dokdonia* sp. MED134, Flavobacterium BAL38). The number of glycosyl hydrolases is based on hits to specific PFAMs in the CAZy database (http://www.cazy.org/) targeting glycosyl hydrolases and polysaccharide lyases. The number of carbohydrate-binding domains (CBM) is also based on hits to PFAMs in the CAZy database. Only matches with E<10^−4^ were considered. The number of peptidases is based on matches to peptidase specific PFAMs (E<10^−4^). The number of TonB channels is based on the presence of a TonB-dependent receptor plug domain (PF07715) and a TonB dependent receptor (PF00593) on the same peptide.
(0.06 MB PDF)

Table S4 Putative sugar uptake and degradation pathways in MS024-2A.
(0.05 MB PDF)

Table S5 Genes and domains with a potential role in adhesion.
(0.05 MB PDF)
