# Supplementary figures and images for: Assembling the Marine Metagenome, One Cell at a Time
Source: PLoS One. 2009 Apr 23;4(4):e5299. doi: 10.1371/journal.pone.0005299 (PMC2668756; doi:10.1371/journal.pone.0005299)

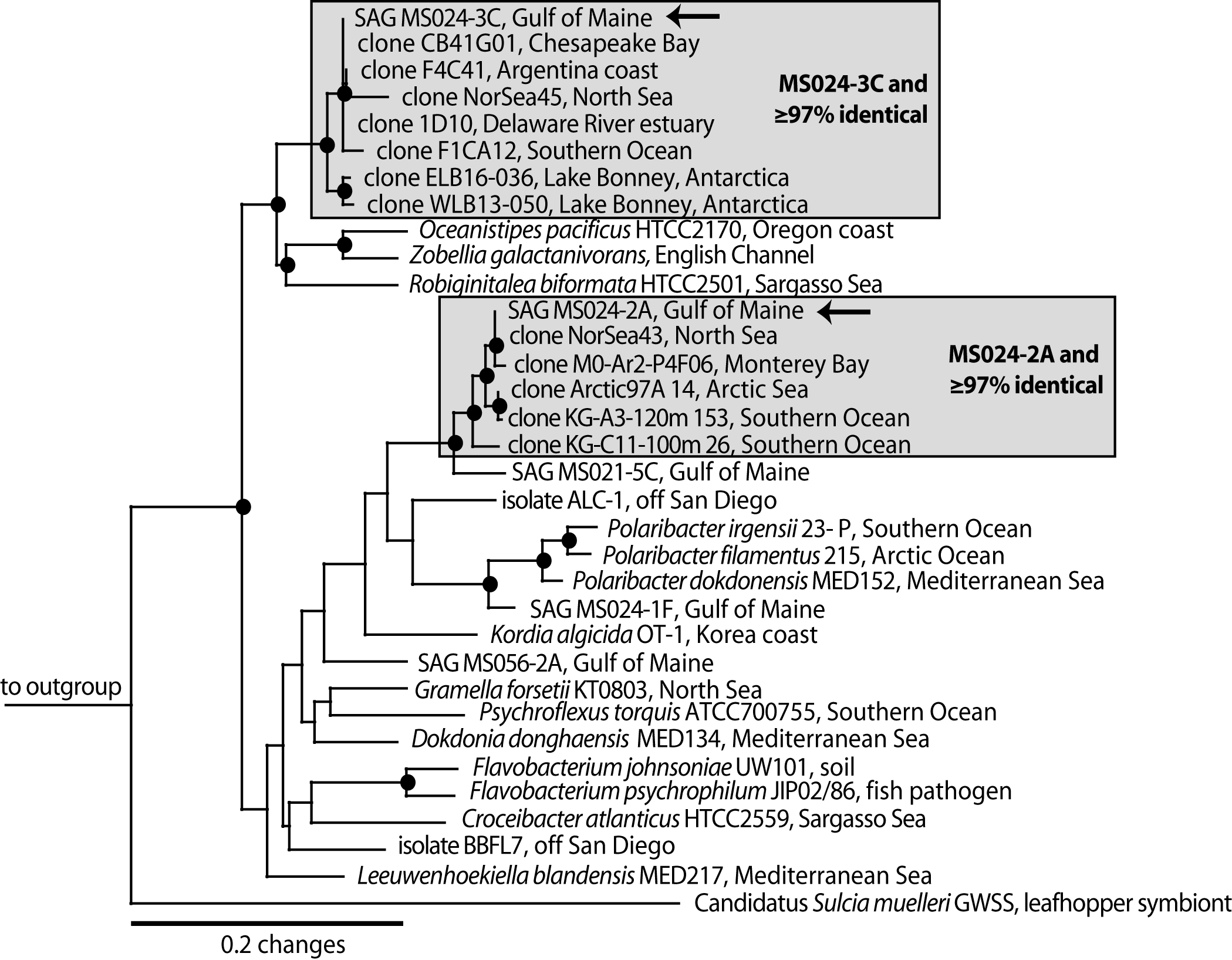

Supplement: Figure S1 — Maximum likelihood phylogenetic tree of 16S rRNA genes. Included are Flavobacteria isolates undergoing whole genome sequencing, single amplified genomes (SAGs) from the same environmental sample as MS024-2A and MS024-3C, as well as those sequences in Genbank that are ≥97% identical to MS024-2A and MS024-3C. Black circles indicate ≥70% neighbor-joining bootstrap support. (0.31 MB TIF) [file pone.0005299.s001.tif]

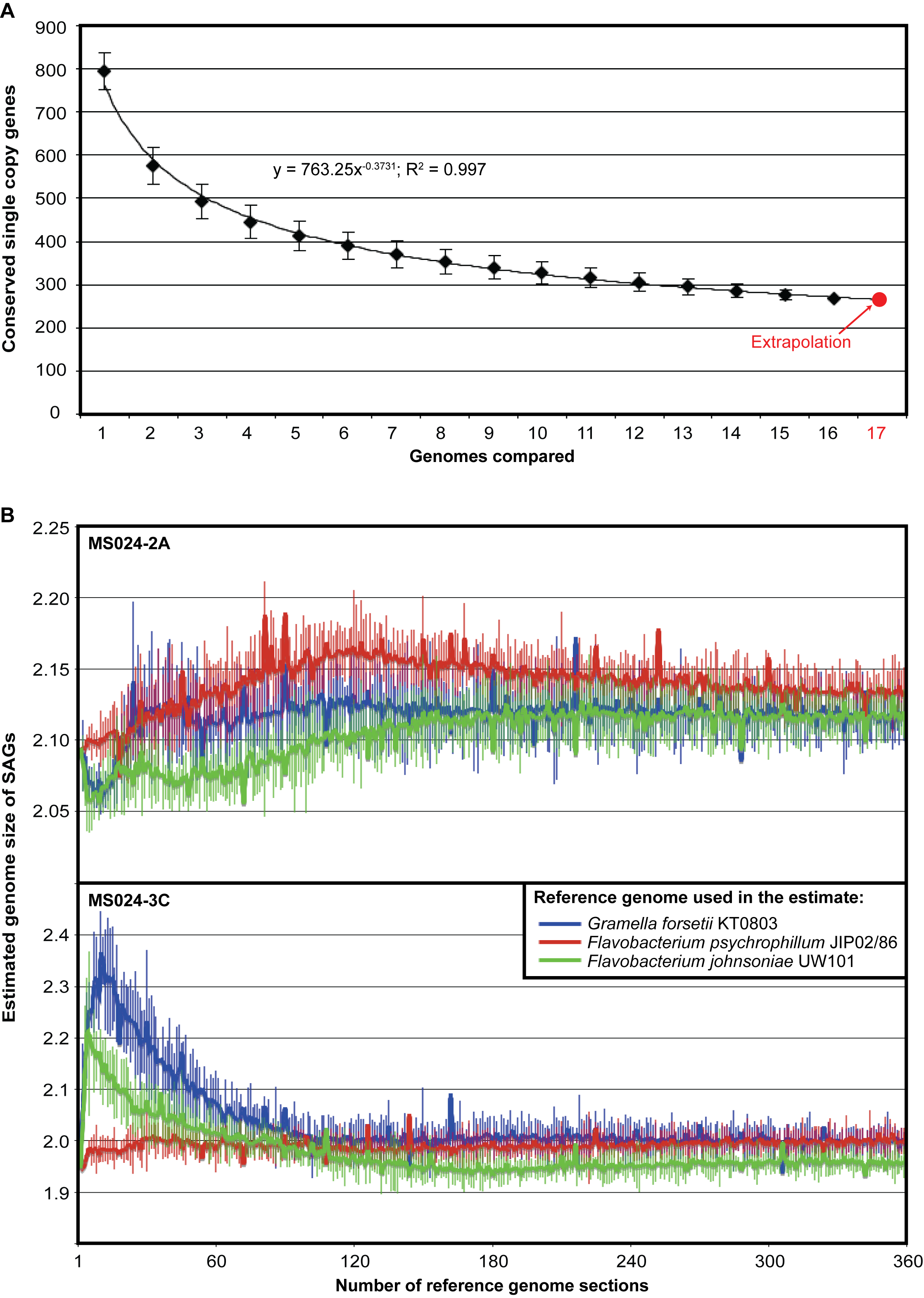

Supplement: Figure S2 — Genome size estimates. A: Number of conserved single copy genes (CSCGs) in Flavobacteria family genomes. The pre-computed COG function distribution from 16 genomes was retrieved from the IMG database, and the number of SCSGs was calculated through an iterative re-sampling of these genomes (see Materials and Methods). Provided are means and standard deviations. A power function fit was applied to the relationship between the count of genomes and the count of CSCGs. This function fit was extrapolated to predict the number of SCGS if a 17th genome was added. B: The effect of CSCG clustering in reference genomes on the estimates of MS024-2A and MS024-3C genome sizes. The genomes of Gramella forsetii KT0803, Flavobacterium johnsoniae UW101 and Flavobacterium psychrophilum JIP02/86 were sequentially divided into various numbers of equal-sized segments, from 1 to 360 per genome. The segmentation was repeated 18 times for each genome, by rotating the segmentation at 20° increments. Genome sizes of SAGs were then estimated based on the recovery of genes representing each of these reference genome segments, as follows: [Σn(SCSCG/TCSCG)] * 0.98/n where TCSCG is the total number of CSCGs in a given reference genome segment; SCSCG is the number of those genes recovered in a SAG, n is the total number of segments, and 0.98 is the correction coefficient to compensate for the expected lower number of CSCGs shared by 17 relative to 16 genomes (Fig. S2A). Provided are means and standard deviations for estimates prepared by rotating the reference genome at 20° increments. (1.89 MB TIF) [file pone.0005299.s002.tif]

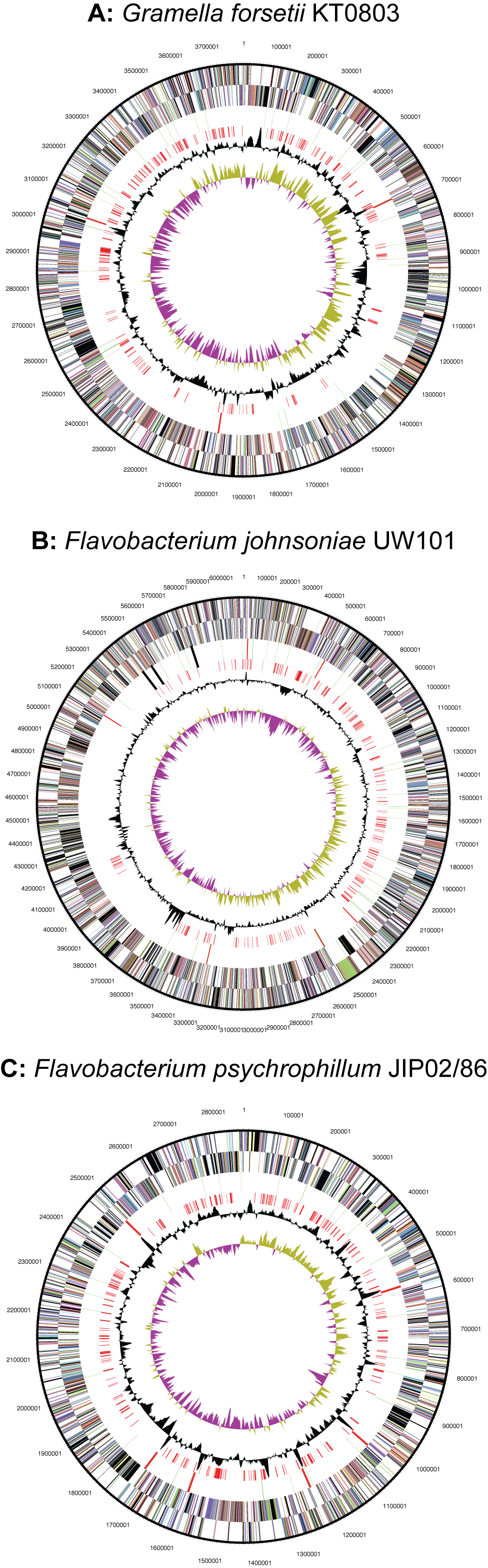

Supplement: Figure S3 — Locations of 268 single copy genes, conserved among Flavobacteria (CSCGs), on the genomes of Gramella forsetii KT0803 (A), Flavobacterium johnsoniae UW101 (B), and Flavobacterium psychrophilum JIP02/86 (C). The outermost two circles indicate start sites of genes and assigned functional categories: forward-strand gene products (circle 1) and reverse-strand gene products (circle 2). Circle 3 indicates RNA genes (tRNAs green, sRNAs red, other RNAs black); circle 4 indicates CSCGs; circle 5 indicates G+C content; circle 6 indicates GC skew (G−C/G+C, khaki are values >1, purple are values <1). Colors of the two outermost circles represent the following functional categories: amino acid biosynthesis, cyan; biosynthesis of cofactors, brown; cell envelope, light gray; cellular processes, light blue; central intermediary metabolism, dark salmon; energy metabolism, green; fatty acid and phospholipid metabolism, orange; other categories, salmon; protein fate, dark gray; purines, pyrimidines, nucleosides, and nucleotides, light green; regulatory functions, light salmon; replication, blue; transcription and translation, magenta; transport and binding proteins, yellow; unassigned, black; unknown function, red. (1.76 MB TIF) [file pone.0005299.s003.tif]

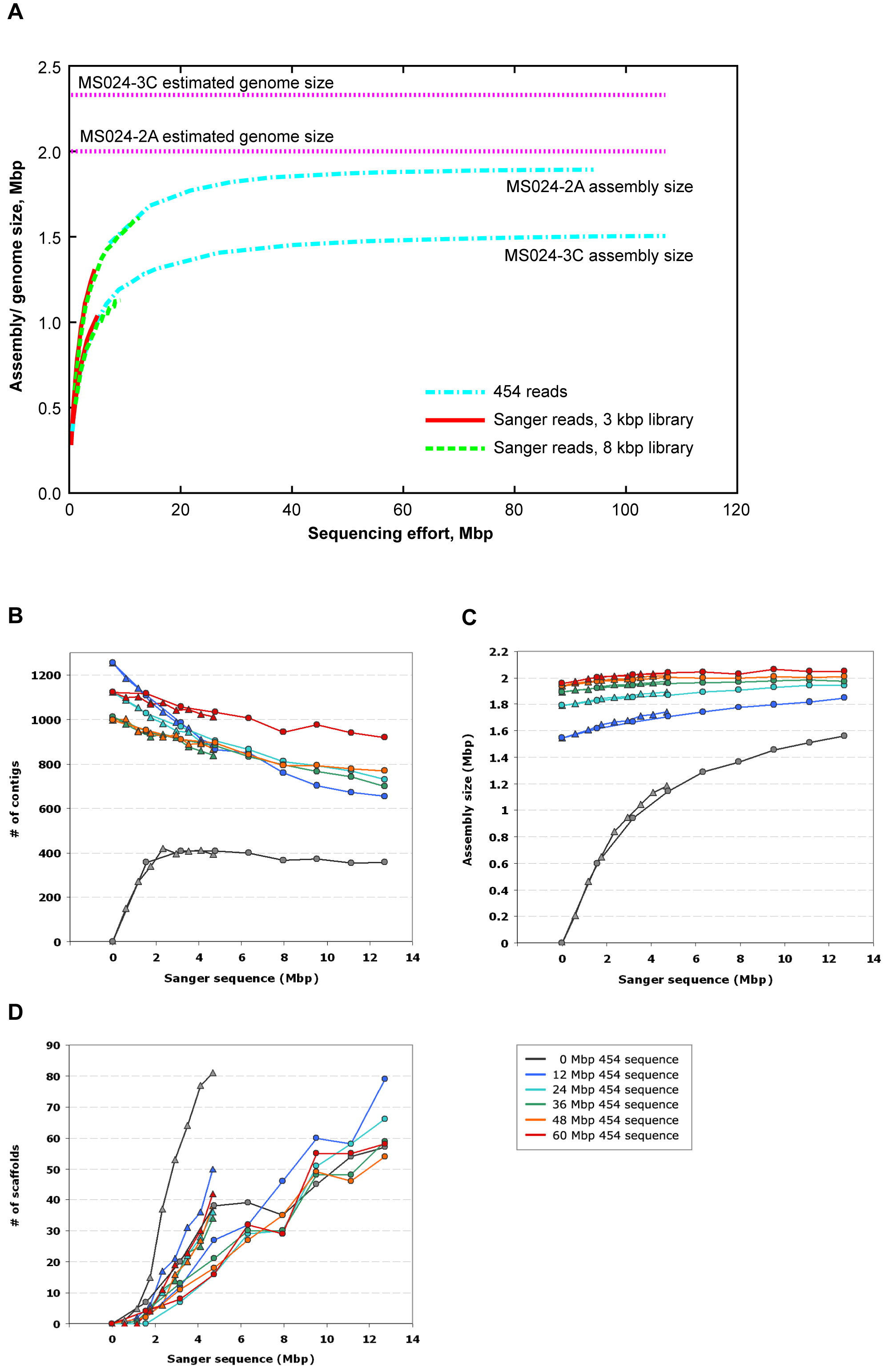

Supplement: Figure S4 — MS024-2A and MS024-3C genome coverage as a function of the sequencing effort (A); and the impact of 454 and Sanger sequence on the number of contigs (B), assembly size (C) and the number of scaffolds (D) for MS024-2A. For panel A, genome size estimates were based on conserved single copy gene (CSCG) analysis (see Materials and Methods). The curves display near-saturation, indicating that additional sequencing would mostly result in repeated sampling of the over-amplified genomic regions and not target the yet missing regions of the genomes. PCR amplification allowed the recovery of some of the missing sequence, suggestive of the under-representation but not lack of these regions. For panels B–D, up to ∼5 Mbp of 3 Kbp library Sanger sequence and ∼13 Mbp of 8 Kbp library Sanger sequence were randomly selected and assembled with 0–60 Mbp of randomly selected pyrosequence to evaluate the assembly outcome. Triangles represent 3 Kbp library sequence and circles represent 8 Kbp library sequence. Our data suggests that increased paired-end Sanger sequence reduced the number of contigs, bringing the assembly together. There was no apparent difference in the impact of 3 Kbp and 8 Kbp clones on the assembly. Adding 454 sequences to the Sanger reads was highly beneficial. Increasing amounts of 454 sequences (most notable at 60 Mbps) raised the number of contigs, which is likely attributed to the biased DNA representation: high amount of pyrosequence begins uncovering some of the under-represented genome regions, creating new contigs. The number of scaffolds kept rising with any added sequence, which is likely attributed to insufficient coverage. The data in panels B–D is based on uncurated pga (v2.6.2) assemblies. The assembly size in panel C thus differs from the extensively curated MS024-2A draft genome assembly size in panel A and Table 1. (0.93 MB TIF) [file pone.0005299.s004.tif]

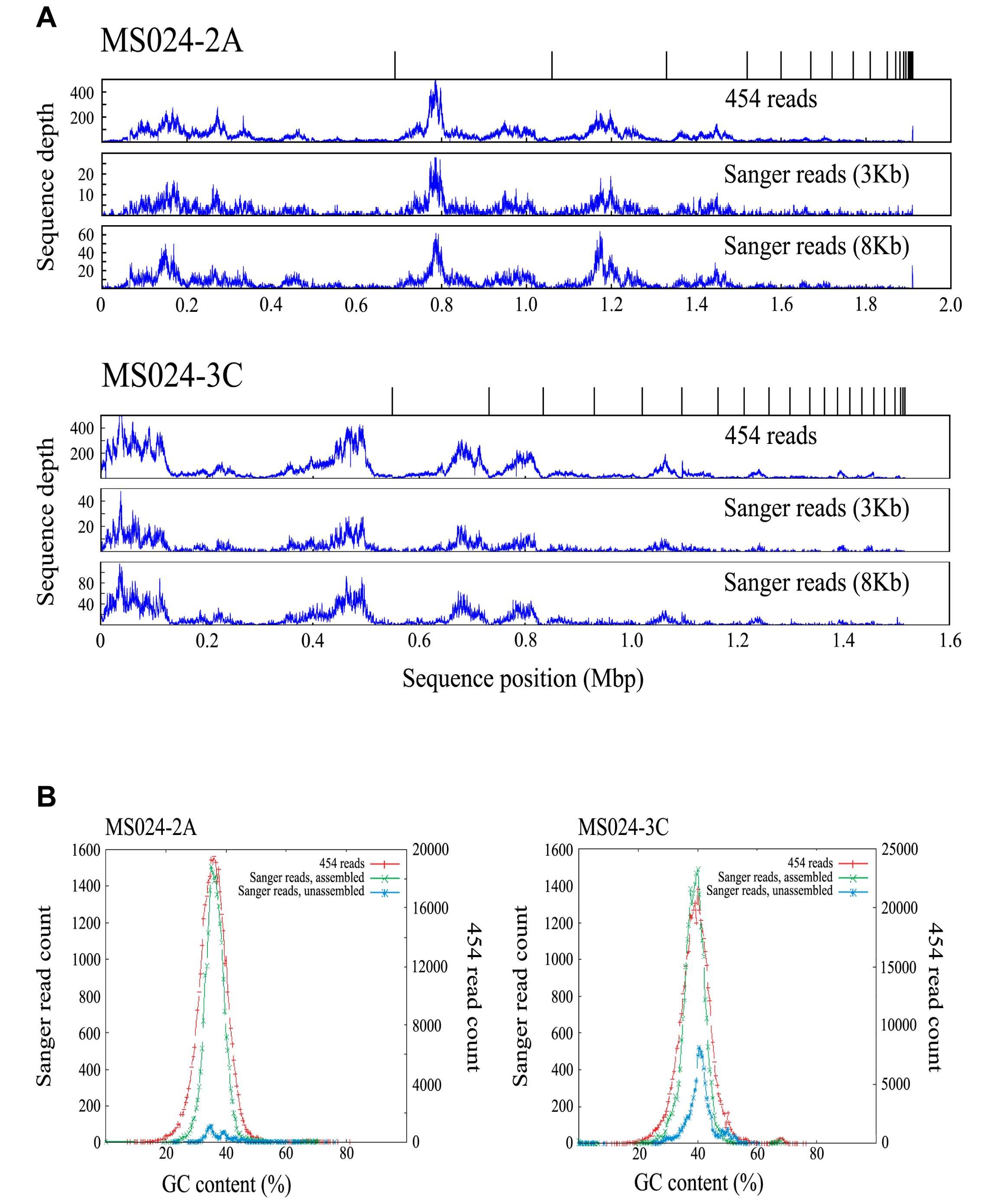

Supplement: Figure S5 — Multiple displacement amplification bias (A) and GC content (B) in MS024-2A and MS024-3C shotgun sequence products. Significant MDA bias is evident from the sequence depth distribution plots for MS024-2A and MS024-3C (A). The contigs for the SAGs were aligned by length and contig breaks, indicated by the tic marks along each top panel. The GC content of the MS024-2A and MS024-3C Sanger sequence and pyrosequence reads demonstrate tight, unimodal distribution at 36% and 39%, suggesting that the reads originate from single phylotypes (B). Contamination with genomes of the same GC contents would, however, be undetectable. (1.22 MB TIF) [file pone.0005299.s005.tif]

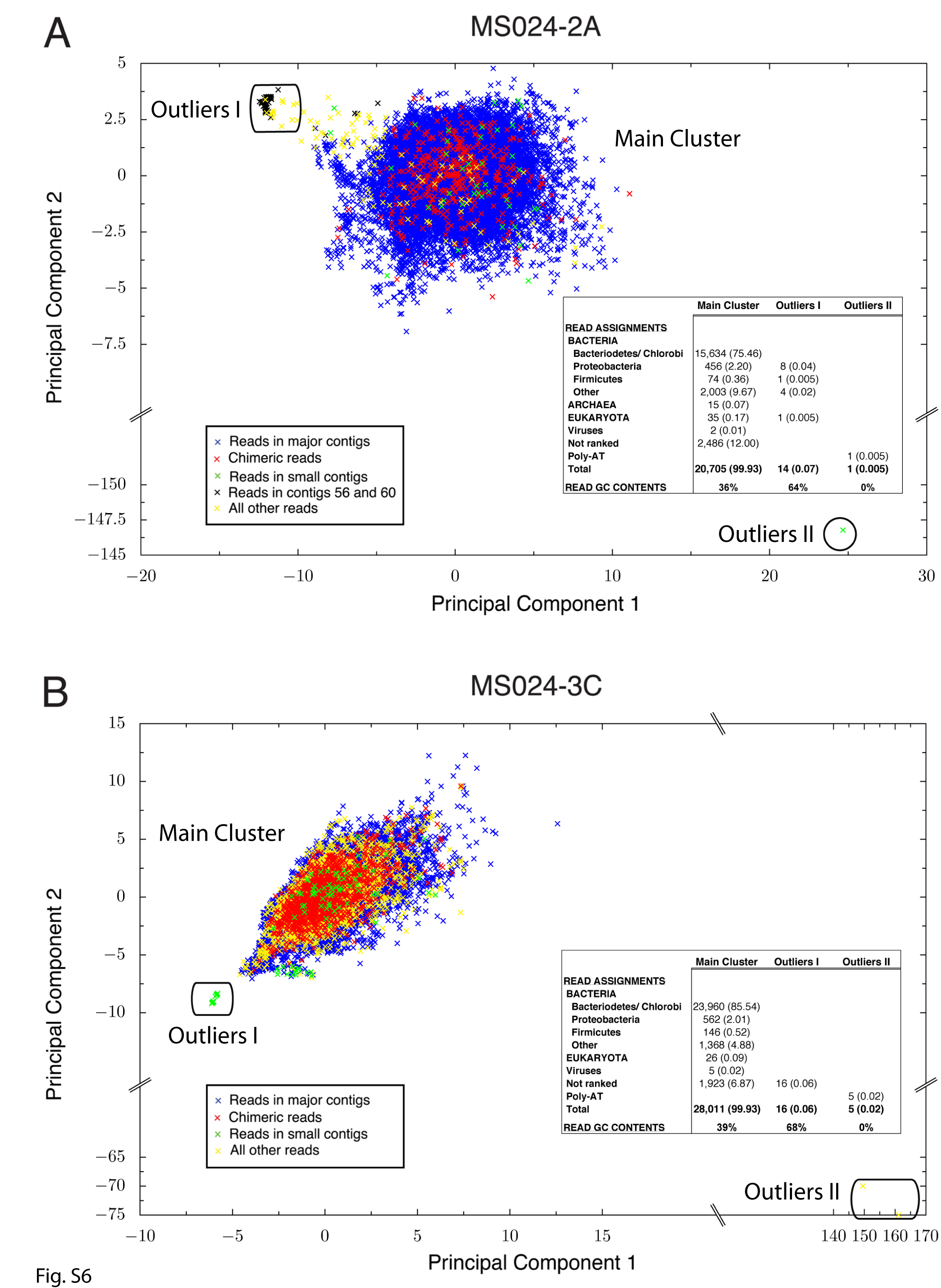

Supplement: Figure S6 — Principal component analysis of nucleotide tetramer frequency in Sanger reads of MS024-2A (A) and MS024-3C (B). The taxonomic origins of the reads were inferred by blastx against GenBank nr database and summarized by MEGAN. Outliers I (0.7% of all reads) were identified as proteobacterial contamination, which may have been introduced during the sequencing process. Outliers II (0.02% of all reads) were identified as polyAT sequence, possibly derived through a buildup of random hexamers. Using simulated datasets from different species of the same GC contents, we were able to separate the genome sequences as based on tetramer signatures (data not shown). The integration of tetramer frequency, blast, and GC content analyses enabled accurate detection of the low levels of contaminating DNA in the shotgun libraries. (1.82 MB TIF) [file pone.0005299.s006.tif]

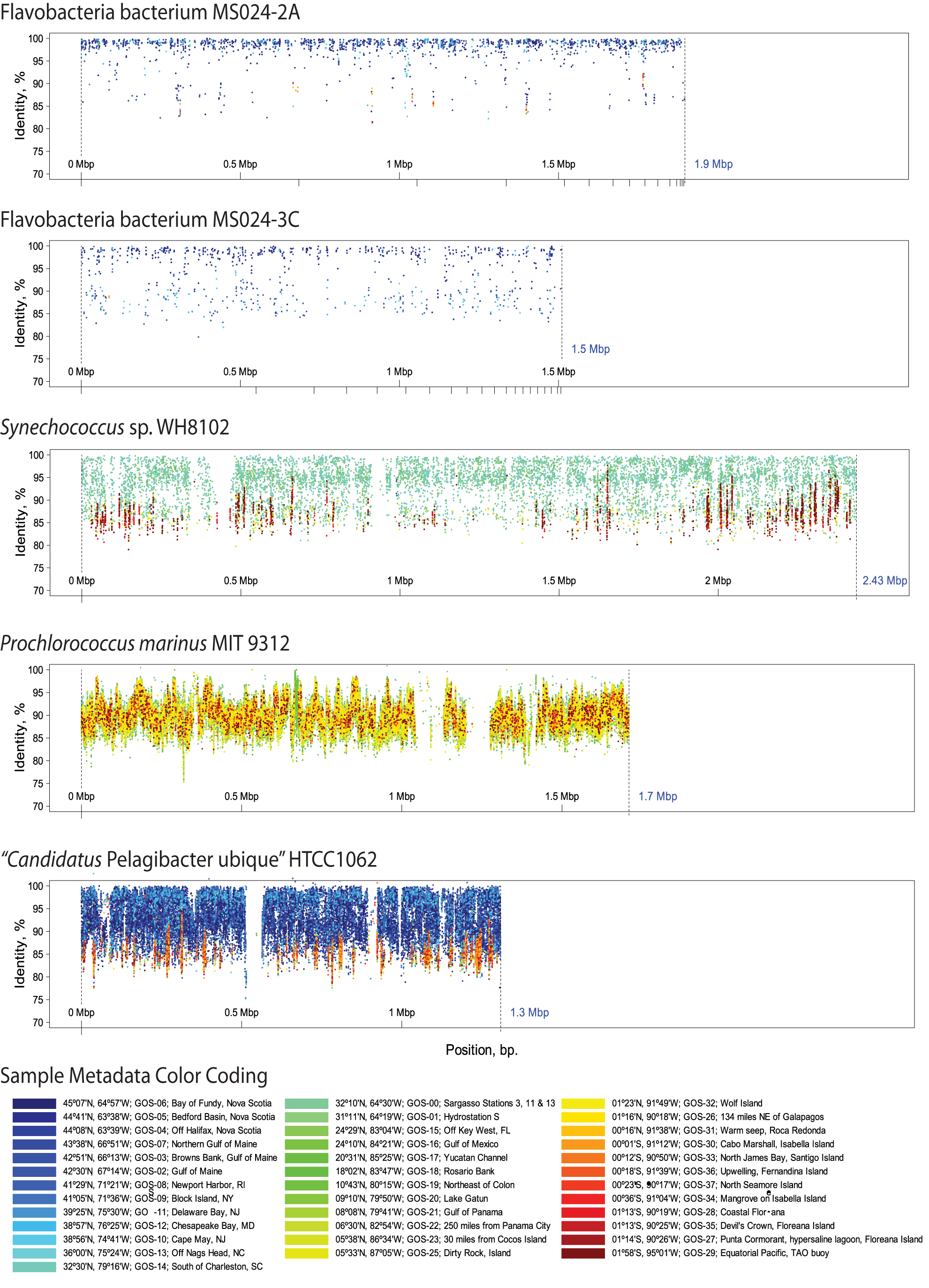

Supplement: Figure S7 — Global Ocean Sampling metagenome fragment recruitment by MS024-2A and MS024-3C and the three best GOS fragment recruiters: Synechococcus sp. WH8102, Prochlorococcus marinus strain MIT 9312 and 9312 and “Candidatus Pelagibacter ubique” HTCC1062. Fragment recruitment was performed with MUMMER and only ≥400 bp alignments were counted. For the two SAGs, the contigs are arranged by length along the x-axis, as indicated by the tick marks. (1.58 MB TIF) [file pone.0005299.s007.tif]

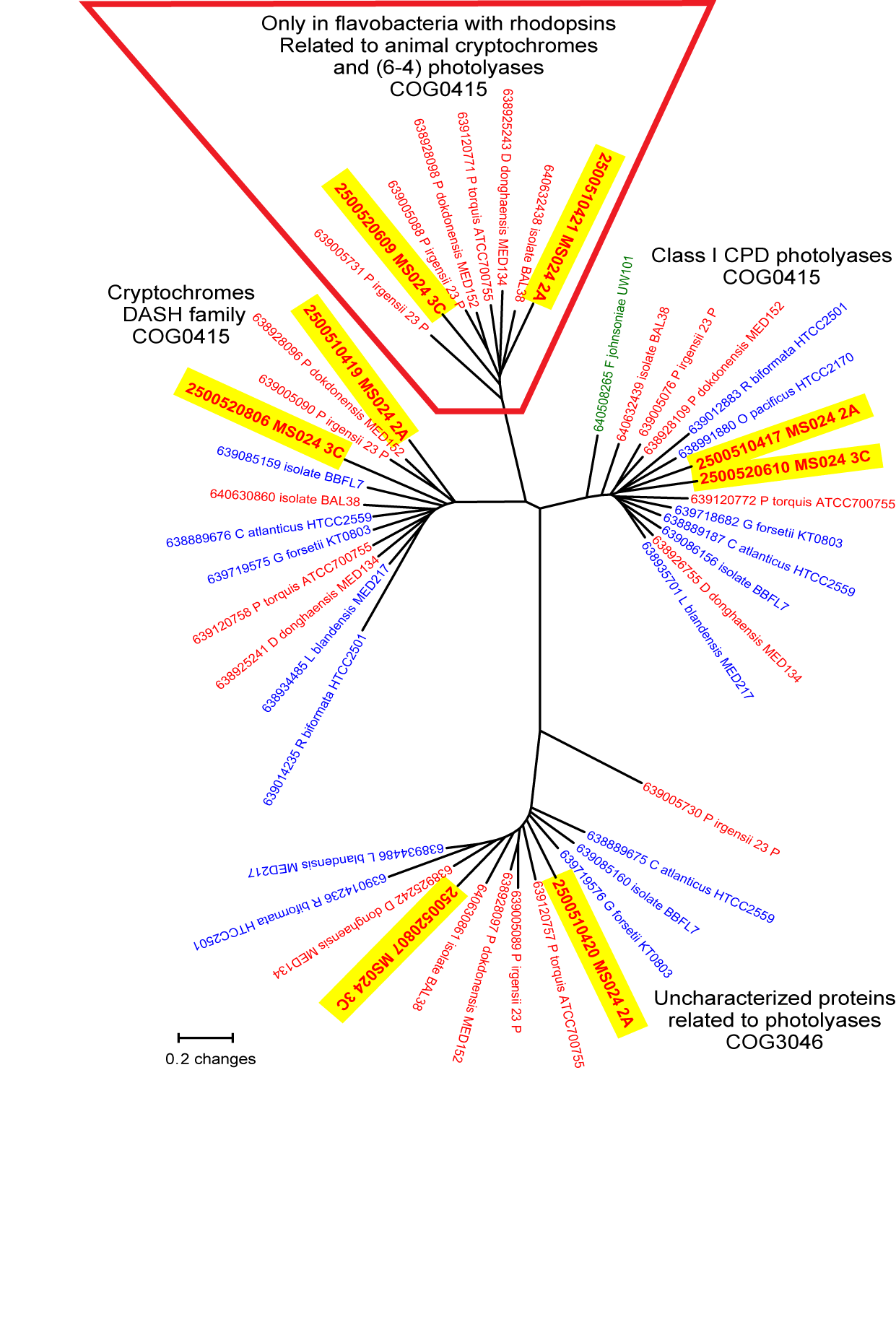

Supplement: Figure S8 — Neighbor-joining tree of DNA photolyase-like genes from all available Flavobacteria genomes. Indicated are IMG gene object identifiers and strain or SAG names. Colors represent marine organisms with rhodopsins (red), marine organisms without rhodopsins (blue), and non-marine organisms (green). (0.40 MB TIF) [file pone.0005299.s008.tif]

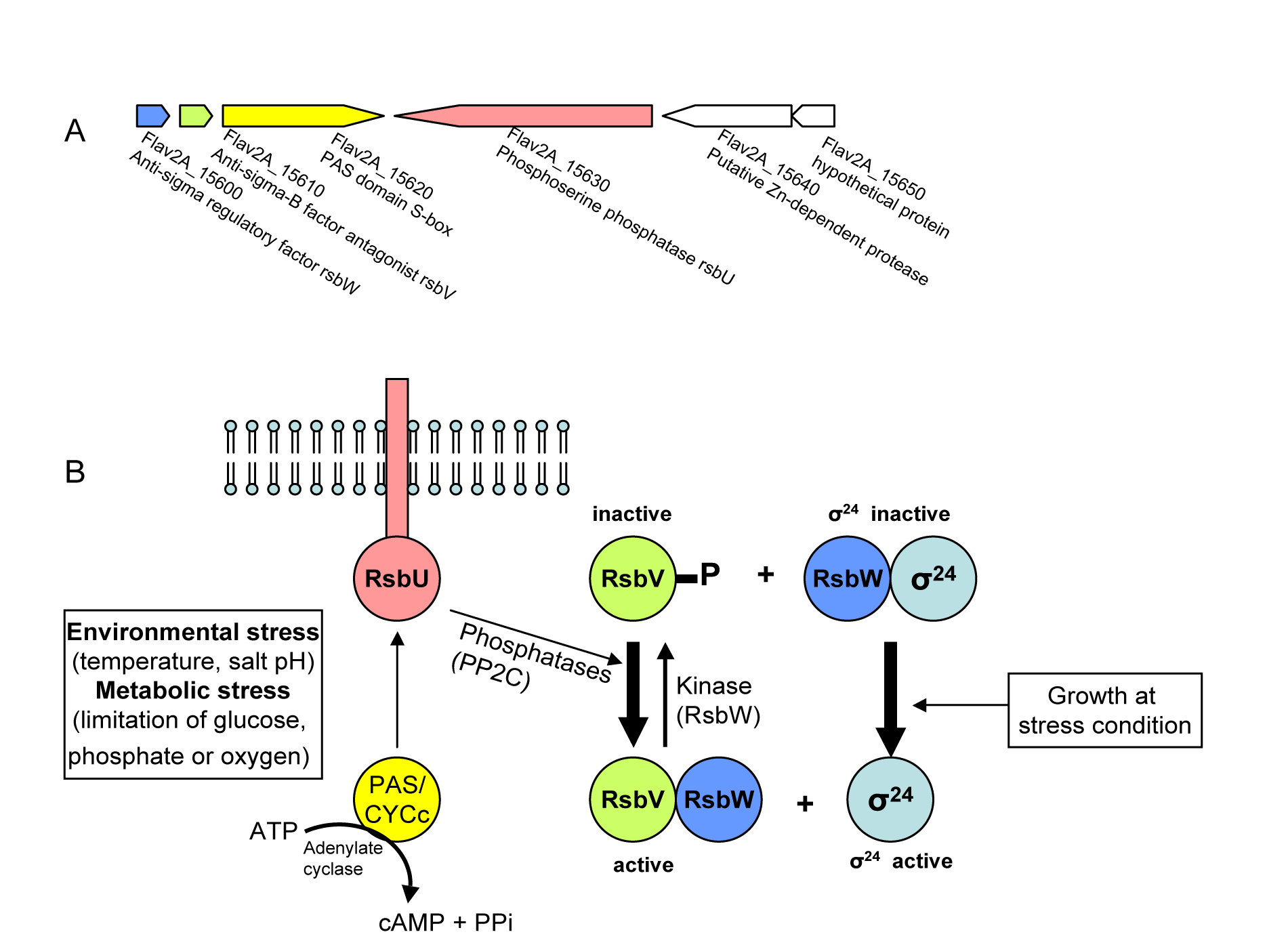

Supplement: Figure S9 — The structure of the rsb operon in MS024-2A (A) and a model for the regulation of σ24 in MS024-2A (B). In panel B, σ24 is held inactive in unstressed MS024-2A as a complex with an anti-sigma factor RsbW. The σ24 is freed from RsbW when a release factor, RsbV, binds to RsbW. In other words, RsbW forms mutually exclusive complexes with either the RsbV protein or σ24. In an unstressed cell, RsbV is inactive due to an RsbW-catalyzed phosphorylation (RsbV-P). Physical stress activates an RsbV-P phosphatase RsbU, which reactivates RsbV. Upon exposure to stress, the putative transmembrane RsbU phosphatase is activated either by a signal at its N-terminal domain or PAS sensory domains of upstream adenylate cyclase. (0.40 MB TIF) [file pone.0005299.s009.tif]
