# Supplementary material for: Assembling the Marine Metagenome, One Cell at a Time
Source: PLoS One. 2009 Apr 23;4(4):e5299. doi: 10.1371/journal.pone.0005299 (PMC2668756; doi:10.1371/journal.pone.0005299)
Supplement: Table S1 — Chimeric rearrangements in SAG DNA. To identify chimeric reads and clones, reads were Q20 quality trimmed and Blast-aligned against the SAG draft assemblies with an alignment minimum of 25 bp. On average, we detected one chimera per 13–27 Kbp of single cell whole genome multiple displacement amplification products. No notable reduction in chimeric rearrangements was detected in the S1-treated DNA samples. (1.27 MB PDF) [file pone.0005299.s010.pdf]

| | MS024-2A | | MS024-3C | |
|---|---|---|---|---|
| | chimeric reads/ clones (%) | overall chimerism | chimeric reads/ clones (%) | overall chimerism |
| **Read-based chimerism** | | | | |
| 3Kb library reads (untreated MDA DNA) | 1.9 | 1 chimer/ 28 Kbp | NA | NA |
| 3Kb library reads (S1 treated MDA DNA) | 2.0 | 1 chimer/ 25 Kbp | 1.9 | 1 chimer/ 33 Kbp |
| 8Kb library reads (S1 treated MDA DNA) | 2.1 | 1 chimer/ 30 Kbp | 1.6 | 1 chimer/ 40 Kbp |
| 454 reads (S1 treated MDA DNA) | | 1 chimer/ 19 Kbp | | 1 chimer/ 25Kbp |
| **Average (all reads)** | | **1 chimer/ 21 Kbp** | | **1 chimer/ 27Kbp** |
| **Clone-based chimerism** | | | | |
| 3Kb clones (untreated MDA DNA) | **14.5** | 1 chimer/ 20 Kbp | **NA** | NA |
| paired reads facing into the same direction | 8.6 | | | |
| paired reads facing away from insert | 3.8 | | | |
| paired reads outside the insert size range | 0.2 | | | |
| paired reads in different contigs | 0.8 | | | |
| paired reads contained in each other | 1.1 | | | |
| 3Kb clones (S1 treated MDA DNA) | **16.8** | 1 chimer/ 15 Kbp | **16.4** | 1 chimer/ 20 Kbp |
| paired reads facing into the same direction | 7.8 | | 9.6 | |
| paired reads facing away from insert | 3.0 | | 0.9 | |
| paired reads outside the insert size range | 3.2 | | 4.5 | |
| paired reads in different contigs | 2.0 | | 0.8 | |
| paired reads contained in each other | 0.8 | | 0.6 | |
| 8Kb clones (S1 treated MDA DNA) | **36.4** | 1 chimer/ 17Kbp | **29.5** | 1 chimer/ 27Kbp |
| paired reads facing into the same direction | 22.6 | | 17.4 | |
| paired reads facing away from insert | 2.5 | | 2.7 | |
| paired reads outside the insert size range | 7.4 | | 3.9 | |
| paired reads in different contigs | 3.3 | | 3.8 | |
| paired reads contained in each other | 0.6 | | 1.8 | |
