# Supplementary material for: Assembling the Marine Metagenome, One Cell at a Time
Source: PLoS One. 2009 Apr 23;4(4):e5299. doi: 10.1371/journal.pone.0005299 (PMC2668756; doi:10.1371/journal.pone.0005299)
Supplement: Table S2 — Key enzymes and metabolic pathways in the uptake and metabolism of N, P, S and Fe. Two pathways for ammonium assimilation were detected in MS024-3C, Gln synthetase (GS)/Glu synthase (GOGAT), and Glu dehydrogenase (GDH) pathways. MS024-2A only contains the GDH pathway. Genes involved in nitrate or nitrite utilization were not found. Polyphosphate kinase catalyzes the formation of polyphosphate granules from ATP. H+-translocating pyrophosphatase couples the energy of PPi hydrolysis to H+ movement across the membrane. Polyphosphate is also a source of PPi. Sulfate assimilation genes involved in the reduction of sulfate to H2S (cysDHNIJE) could not be detected. The sole source of sulfur appears to be organic material or H2S. Most of the peptides involved in iron metabolism are transporters. TonB-dependent outer membrane channels are mainly known for the transport of iron in Gram-negative bacteria. (0.07 MB PDF) [file pone.0005299.s011.pdf]

	MS024-2A ORFs	MS024-3C ORFS
**Nitrogen assimilation**		
Ammonium channel (*amtB*)		Flav3_or0599
Glutamate synthase	Flav2a_or1206, (*gltB*), Flav2a_or1205, (*gltD*)	Flav3c_or0884 (*gltB*), Flav3c_or0885 (*gltD*)
Nitrogen regulatory protein P-II		Flav3c_or0600
Glutamine synthetase, type II		Flav3c_or0588
Glutamine synthetase, type III		Flav3c_or0589
Glutamate dehydrogenase	Flav2a_or0639	Flav3c_or1126
Allophanate hydrolase	Flav2A_or0245, Flav2A_or0246	
**Phosphate metabolism**		
Phosphate permease	Flav2A_or0989	Flav3C_or0330
Polyphosphate kinase	Flav2A_or1736	Flav3C_or0433
Exopolyphosphatase	Flav2A_or1737	
H^+-translocating pyrophosphatase	Flav2A_or1260	Flav3C_or0715
Soluble pyrophosphatase		Flav3C_or0714
**Sulfur assimilation**		
Sulphate permease	Flav2A_or0259, Flav2A_or0804, Flav2A_or1153	Flav3C_or1133, Flav3C_or1154
APS/PAPS pathway	no *cysDHNIJE*	no *cysDHNIJE*
Cysteine synthase (*cysK* or *cysM*)	Flav2A_or0331, Flav2A_or1471	Flav3C_or0269
**Iron assimilation**		
Mn^{2+}/Fe^{2+} transporter, NRAMP family	Flav2A_ or1119, Flav2A_or1223	Flav3C_or0145
Ferrous Iron Uptake (FeoB) family transporter	Flav2A_or0937	
Vacuolar Iron Transporter (VIT) family	Flav2A_or0083	
Fur	Flav2A_or0540	Flav3C_or0097
FecR	Flav2A_or1173	
