# Supplementary material for: Assembling the Marine Metagenome, One Cell at a Time
Source: PLoS One. 2009 Apr 23;4(4):e5299. doi: 10.1371/journal.pone.0005299 (PMC2668756; doi:10.1371/journal.pone.0005299)
Supplement: Table S3 — The total and genome size (Mbp)-normalized number of hydrolytic enzymes, TonB dependent/ligand-gated channels and SusD homologs. Included in the analysis are MS024-2A, MS024-3C and other Bacteroidetes genomes with high hydrolytic potential (Flavobacterium johnsoniae UW101, Gramella forsetii KT0803, Cytophaga hutchinsonii ATCC 33406, Bacteroides thetaiotaomicron VPI-5482, Flavobacterium psychrophilum JIP02/86) and proteorhodopsin-containing Bacteroidetes (Polaribacter sp. MED152, Polaribacter irgensii 23-P, Dokdonia sp. MED134, Flavobacterium BAL38). The number of glycosyl hydrolases is based on hits to specific PFAMs in the CAZy database (http://www.cazy.org/) targeting glycosyl hydrolases and polysaccharide lyases. The number of carbohydrate-binding domains (CBM) is also based on hits to PFAMs in the CAZy database. Only matches with E<10−4 were considered. The number of peptidases is based on matches to peptidase specific PFAMs (E<10−4). The number of TonB channels is based on the presence of a TonB-dependent receptor plug domain (PF07715) and a TonB dependent receptor (PF00593) on the same peptide. (0.06 MB PDF) [file pone.0005299.s012.pdf]

| Genome | Genome size (Mbp) | Peptidases | Glycosylases | TonB | SusD | CBM | Sulfatases |
|---|---|---|---|---|---|---|---|
| MS024-2A | 1.9 | 73 / 38.3 | 20 / 10.5 | 16 / 8.4 | 3 / 1.6 | 0 / 0 | 6 / 3.1 |
| MS024-3C | 1.5 | 59 / 38.9 | 12 / 7.9 | 15 / 9.9 | 3 / 2 | 0 / 0 | 1 / 0.7 |
| *Polaribacter* sp. MED152 | 3.0 | 96 / 32.4 | 31 / 10.4 | 30 / 10.1 | 5 / 1.7 | 2 / 0.7 | 3 / 1 |
| *F. johnsoniae* UW101 | 6.1 | 144 / 23.7 | 141 / 23.2 | 94 / 15.5 | 35 / 5.8 | 9 / 1.5 | 12 / 2 |
| *G. forsetii* KT0803 | 3.8 | 121 / 31.9 | 48 / 12.6 | 40 / 10.5 | 17 / 4.5 | 3 / 0.8 | 3 / 0.8 |
| *C. hutchinsonii* ATCC 33406 | 4.4 | 92 / 20.8 | 51 / 11.5 | 16 / 3.6 | 3 / 0.7 | 5 / 1.1 | 2 / 0.5 |
| *B. theta.* VPI-5482 | 6.3 | 114 / 18.2 | 245 / 39.1 | 127 / 20.3 | 88 / 14.1 | 30 / 4.8 | 31 / 5 |
| *F. psychrop.* JIP02/86 | 2.9 | 90 / 31.4 | 10 / 3.5 | 25 / 8.7 | 0 / 0 | 0 / 0 | 2 / 0.7 |
| *P. irgensii* 23-P | 2.7 | 95 / 34.6 | 13 / 4.7 | 41 / 14.9 | 10 / 3.6 | 1 / 0.4 | 2 / 0.7 |
| *Dokdonia* sp. MED134 | 3.3 | 120 / 36.3 | 22 / 6.7 | 27 / 8.2 | 4 / 1.2 | 4 / 1.2 | 1 / 0.3 |
| BAL38 | 2.8 | 92 / 32.8 | 25 / 8.9 | 19 / 6.8 | 3 / 1.1 | 1 / 0.4 | 6 / 2.1 |
