# Supplementary material for: Assembling the Marine Metagenome, One Cell at a Time
Source: PLoS One. 2009 Apr 23;4(4):e5299. doi: 10.1371/journal.pone.0005299 (PMC2668756; doi:10.1371/journal.pone.0005299)
Supplement: Table S4 — Putative sugar uptake and degradation pathways in MS024-2A. (0.05 MB PDF) [file pone.0005299.s013.pdf]

Sugar	Import	Degradation	Notes
D-galacturonate	Flav2A_or1522, Flav2A_or0121, Flav2A_or1125	Flav2A_or1676, Flav2A_or0931, Flav2A_or1670, Flav2A_or1675, Flav2A_or1674	A transporter similar to hexuronate porter (ExuT) for likely uptake of D-galacturonate from the marine environment
Galactose	Flav2A_or0697, Flav2A_or0939	Flav2A_or0670, Flav2A_or0565	Almost complete Leloir pathway
Gluconate	Flav2A_or0182, Flav2A_or0632	Flav2A_or0691, Flav2A_or0692	Unique gluconate kinase
Sucrose	PTS: Flav2A_or0718, Flav2A_or0719, Flav2A_or0720, Flav2A_or0721, Flav2A_or0722, Flav2A_or1139	Flav2A_or0208, Flav2A_or0878, Flav2A_or1128, Flav2A_or1675, Flav2A_or1688	Complete sucrose degradation pathway and PTS for sucrose uptake
Xylose	Flav2A_or0207, Flav2A_or0924, Flav2A_or1021	Flav2A_or1085, Flav2A_or1084,	Complete xylose degradation pathway
Trehalose	PTS: Flav2A_or0718, Flav2A_or0719, Flav2A_or0720, Flav2A_or0721, Flav2A_or0722, Flav2A_or1139	Flav2A_or0410, Flav2A_or0411	Complete trehalose degradation pathway and PTS for its uptake
Mannitol	PTS: Flav2A_or0718, Flav2A_or0719, Flav2A_or0720, Flav2A_or0721, Flav2A_or0722, Flav2A_or1139	Flav2A_or0931	Can use mannitol as carbon source, has PTS is for mannitol uptake
