# Supplementary material for: Assembling the Marine Metagenome, One Cell at a Time
Source: PLoS One. 2009 Apr 23;4(4):e5299. doi: 10.1371/journal.pone.0005299 (PMC2668756; doi:10.1371/journal.pone.0005299)
Supplement: Table S5 — Genes and domains with a potential role in adhesion. (0.05 MB PDF) [file pone.0005299.s014.pdf]

Domain	Protein family	MS024-2A ORFs	MS024-3C ORFs
Thrombospondin type 3 repeat	PF02412	Flav2A_or0633, Flav2A_or0725, Flav2A_or0731, Flav2A_or0882, Flav2A_or0902, Flav2A_or1065, Flav2A_or1073, Flav2A_or1114, Flav2A_or1214, Flav2A_or1405, Flav2A_or1758	Flav3C_or0249, Flav3C_or0584, Flav3C_or1101, Flav3C_or1311
Cadherin domain	PF00028	Flav2A_or0708, Flav2A_or0725, Flav2A_or1114, Flav2A_or1758	Flav3C_or1311
Fibronectin type III	PF00041	Flav2A_or0724, Flav2A_or1065, Flav2A_or1066, Flav2A_or1472, Flav2A_or1475	Flav3C_or0249, Flav3C_or0250
PKD	PF00801	Flav2A_or1472, Flav2A_or1474	Flav3C_or0249, Flav3C_or1331
FG-GAP	PF01839	Flav2A_or0506, Flav2A_or0708, Flav2A_or0711, Flav2A_or0713, Flav2A_or0902, Flav2A_or1019	Flav3C_or0457, Flav3C_or1026, Flav3C_or1028, Flav3C_or1029
Hep_Hag	PF05658	Flav2A_or0726	
ASPIC and UnbV	PF07593	Flav2A_or1019	Flav3C_or1026, Flav3C_or1028, Flav3C_or1029
HYR	PF02494	Flav2A_or1073, Flav2A_or1345	Flav3C_or1311
Leu rich repeat	PF00560	Flav2A_or0688	
Cellulose binding motif	PF00553	Flav2A_or1423, Flav2A_or1472	
Von Willebrand factor type A	PF00092	Flav2A_or0856, Flav2A_or0858, Flav2A_or0859, Flav2A_or0999	Flav3C_or0198, Flav3C_or1148, Flav3C_or1149
Cna protein B-type	PF05738	Flav2A_or0990	Flav3C_or1044, Flav3C_or1331
Putative Ig domain	PF05345	Flav2A_or1214	
Bacterial Ig-like domain (group 1)	PF02369		Flav3C_or1099
Bacterial Ig-like domain (group 3)	PF07523	Flav2A_or1214, Flav2A_or1345	
Laminin G domain	PF02210	Flav2A_or1758	
F5/8 type C domain	PF00754	Flav2A_or0035	
GA module	PF01468		Flav3C_or0206
PA14 domain	PF07691		Flav3C_or1311, Flav3C_or1331
Fasciclin	PF02469		Flav3C_or1216
